# Commentary on Gut Microbiome–Metabolome Alterations in Advanced Parkinson's Disease With Motor Complications

**DOI:** 10.1002/cns.70789

**Published:** 2026-02-23

**Authors:** Hamza Rashid

**Affiliations:** ^1^ Khyber Medical University Peshawar Pakistan

To the Editor,

1

We read with great interest the recent article by Qian et al. exploring fecal microbiome dysbiosis and plasma metabolomic alterations in patients with advanced Parkinson's disease (PD) with motor complications (PD‐MC) [1]. The authors should be commended for applying an integrated multi‐omics strategy across independent cohorts, offering valuable insights into gut–brain axis perturbations in later stage PD.

While the study provides important associative findings, several methodological considerations merit discussion and may help refine interpretation of the results and guide future research.

First, the cross‐sectional design inherently limits causal inference. Gut microbiota composition and circulating metabolites are dynamic and influenced by disease duration, aging, and therapeutic exposure. As a result, it remains unclear whether the observed dysbiosis contributes to the development of motor complications or represents a downstream consequence of advanced disease and long‐term dopaminergic therapy. Longitudinal studies tracking microbiome–metabolome changes across disease stages would be particularly informative in clarifying these temporal relationships [2, 3].

Second, medication‐related confounding warrants closer attention. Patients with PD‐MC are typically exposed to heterogeneous dopaminergic regimens, including variable levodopa equivalent daily doses and adjunct therapies such as COMT or MAO‐B inhibitors. These treatments are known to influence gastrointestinal motility, microbial composition, and metabolic pathways, potentially shaping both fecal microbiota and plasma metabolomic profiles independent of disease biology [4]. More granular adjustment or stratified analyses based on medication burden could therefore strengthen causal interpretation.

Third, dietary intake represents a major determinant of gut microbiota structure and function. Although certain confounders were addressed through exclusion criteria, quantitative dietary variables—such as fiber intake, protein consumption, and fermented food exposure—were not deeply incorporated into the analytical models. Integration of standardized dietary assessments may further refine microbiome–metabolome associations and reduce residual confounding [2].

Additionally, functional interpretation of microbiome alterations relied largely on taxonomic inference derived from 16S rRNA sequencing. While appropriate for community profiling, this approach provides limited insight into microbial metabolic capacity. The integration of shotgun metagenomics or fecal metabolomics in future studies could directly validate functional pathways, particularly those related to short‐chain fatty acid production and amino acid metabolism, which are highly relevant to neuroinflammation and neurodegeneration [3, 5].

Another important consideration concerns the interpretation of plasma metabolomic findings. Circulating metabolite levels do not necessarily reflect central nervous system exposure due to the modulatory role of the blood–brain barrier and peripheral metabolism. Consequently, the link between altered plasma metabolites and neuronal dysfunction remains indirect [6]. Complementary cerebrospinal fluid analyses or experimental models may help bridge this translational gap.

Finally, advanced PD represents a biologically heterogeneous condition encompassing diverse motor phenotypes and symptom trajectories. Subgroup analyses based on motor phenotype or non‐motor symptom burden may uncover subtype‐specific gut–brain interactions. Furthermore, as the study population was geographically and ethnically homogeneous, external validation in multi‐ethnic cohorts would enhance generalizability.

In conclusion, Qian et al. provide a valuable contribution to understanding gut microbiome–metabolome alterations in PD with motor complications. Addressing these methodological considerations in future work may further clarify causal mechanisms and accelerate the development of microbiota‐informed biomarkers and therapeutic strategies [2, 6].

## Conflicts of Interest

The author declares no conflicts of interest.

## Data Availability

The author has nothing to report.
